# Rapid Diffusion Magnetic Resonance Imaging Using Slice-Interleaved Encoding

**DOI:** 10.1016/j.media.2022.102548

**Published:** 2022-07-16

**Authors:** Tiantian Xu, Ye Wu, Yoonmi Hong, Sahar Ahmad, Khoi Minh Huynh, Zhixing Wang, Weili Lin, Wei-Tang Chang, Pew-Thian Yap

**Affiliations:** a Department of Computer Science, University of North Carolina, Chapel Hill, NC 27599, USA; b Department of Biomedical Engineering, University of North Carolina, Chapel Hill, NC 27599, USA; c Department of Biomedical Engineering, University of Virginia, Charlottesville, VA 22904, USA; d Department of Radiology, University of North Carolina, Chapel Hill, NC 27599, USA; e Biomedical Research Imaging Center (BRIC), University of North Carolina, Chapel Hill, NC 27599, USA

**Keywords:** Diffusion MRI, Slice-Interleaved Diffusion Encoding (SIDE), Fast MRI

## Abstract

In this paper, we present a robust reconstruction scheme for diffusion MRI (dMRI) data acquired using slice-interleaved diffusion encoding (SIDE). When combined with SIDE undersampling and simultaneous multi-slice (SMS) imaging, our reconstruction strategy is capable of significantly reducing the amount of data that needs to be acquired, enabling high-speed diffusion imaging for pediatric, elderly, and claustrophobic individuals. In contrast to the conventional approach of acquiring a full diffusion-weighted (DW) volume per diffusion wavevector, SIDE acquires in each repetition time (TR) a volume that consists of interleaved slice groups, each group corresponding to a different diffusion wavevector. This strategy allows SIDE to rapidly acquire data covering a large number of wavevectors within a short period of time. The proposed reconstruction method uses a diffusion spectrum model and multi-dimensional total variation to recover full DW images from DW volumes that are slice-undersampled due to unacquired SIDE volumes. We formulate an inverse problem that can be solved efficiently using the alternating direction method of multipliers (ADMM). Experiment results demonstrate that DW images can be reconstructed with high fidelity even when the acquisition is accelerated by 25 folds.

## 1. Introduction

Diffusion magnetic resonance imaging (dMRI) is a unique imaging technique for characterizing tissue microstructure and neural pathways in the human brain. It has been utilized to study brain disorders such as Alzheimer’s disease, schizophrenia, and mild traumatic brain injury (Shenton et al., 2012; Colgan et al., 2016; Sui et al., 2015; Yuh et al., 2014). Diffusion tensor imaging (DTI) is the simplest form of dMRI and requires diffusion-weighted (DW) images of at least six noncollinear diffusion directions to capture microstructural changes in terms of the magnitude and mode of diffusion anisotropy (Ennis and Kindlmann, 2006; Basser et al., 1994; Basser and Pierpaoli, 2011; Alexander et al., 2007). However, the diffusion tensor model is incapable of resolving multiple fiber orientations within each voxel. High angular resolution diffusion imaging (HARDI) (Tuch et al., 2002; Tuch, 2004) overcomes this limitation by acquiring a few tens of noncollinear gradient directions uniformly distributed on a sphere (Tuch et al., 2003) to capture multiple orientations via the orientation distribution function (ODF). Sensitivity to tissue micostructure can be further improved with multi-shell acquisitions (Jeurissen et al., 2014; Schneider et al., 2017; Zhang et al., 2012), at the cost of prolonged scan times, increasing susceptibility to motion artifacts, and patient discomforts (Christiaens et al., 2021).

Imaging with low spatial resolution is a straightforward means to shorten the acquisition time. However, the associated partial volume effects (PVE), although partially mitigable using multi-compartment models (Roine et al., 2015; Xu et al., 2017), may negatively affect microstructural analysis and fiber tractography (Vos et al., 2011; Huynh et al., 2020). Simultaneous multi-slice (SMS) (Barth et al., 2016) techniques can be used to acquire multiple diffusion-encoded slices simultaneously with high scan efficiency. However, the geometric factor induced noise amplification and residual aliasing artifacts from overlapped slices pose challenges to SMS imaging, limiting acceleration to a factor of 2 ~ 5.

**Table:** 

Algorithm 1. Optimization
$$\begin{array}{cc} \; & \;\mathrm{Require}:\text{Input data}\;\{E_{q}^{{}^{{}_{S}}}\}\\ \; & \;\mathrm{Require}:A,\{H_{l}\},\{w_{ql}\}\\ \; & \;\mathrm{Require}:\text{Parameters}\;\gamma_{x},\gamma_{y},\gamma_{z},\lambda ,\rho_{1},\rho_{2},\varepsilon_{1},\varepsilon_{2},\;\text{and}\;\text{MaxIter}\\ \; & \;\;\;\;\text{Let}\;t=0,\Psi_{q}(t)=0,\Phi (t)=0;\\ \; & \;\;\;\;\mathrm{repeat}\\ \; & \;\;\;\;\;t\leftarrow t+1;\\ \; & \;\;\;\;\;\text{Solve for}\;V\;\text{via}\;(12);\\ \; & \;\;\;\;\;\text{Solve for}\;\{U_{q}{\}}_{q=1}^{N_{Q}}\;\text{via}\;(13);\\ \; & \;\;\;\;\;\text{Solve for}\;E\;\text{via}\;(14);\\ \; & \;\;\;\;\;\text{Update}\;\Psi_{q}\;\text{via}\;(15);\\ \; & \;\;\;\;\;\text{Update}\;\Phi \;\text{via}\;(16);\\ \; & \;\;\;\;\mathrm{until}\;(t=\text{MaxIter})\;\vee \\ \; & \;\;\;\;\;\left(\|\Psi_{q}(t)-\Psi_{q}(t-1){\|}_{2}^{2}\leq \varepsilon_{1}\wedge \|\Phi (t)-\Phi (t-1){\|}_{2}^{2}\leq \varepsilon_{2}\right)\\ \; & \;\;\;\;\mathrm{return}\;E; \end{array}$$

Redundancy in q-space and/or k-space has been exploited to recover DW images from a reduced number of measurements (Ning et al., 2016; Ramos-Llordén et al., 2020). Ning et al. (2016) proposed a q-space undersampling scheme using three sets of intersecting thick slices, each corresponding to different gradient directions. The high-resolution images are then recovered from these thick slices for all gradient directions using joint super resolution and compressed sensing. Ramos-Llordén et al. (2020) proposed a method called gSlider-SR to accelerate high-resolution dMRI acquisition by downsampling in both RF encoding space and q-space. Signal recovery is carried out using spherical ridgelets with compressed sensing (CS) reconstruction.

Machine learning approaches can be used to improve image reconstruction from undersampled data. Zhang et al. (2020) utilized kernel low-rank CS to reconstruct high-resolution DW images from undersampled k-space data, leveraging the correlation between DW images learned using kernel principal component analysis from low-resolution DW images reconstructed from central k-space data. Hong et al. (2019) proposed a slice-interleaved sampling scheme to accelerate dMRI data acquisition and a graph convolutional neural network (GCNN) for image reconstruction. While improving image reconstruction, these methods rely on the availability of sufficient training data.

In this paper, we introduce a robust reconstruction method for highly undersampled SIDE data. SIDE works in tandem with SMS to capture information associated with a wide range of diffusion encodings in a short amount of time. SIDE acquires image slices in an order that is unlike conventional dMRI, allowing better reconstruction particularly with undersampling. In contrast to the conventional approach of acquiring a full DW volume per diffusion wavevector, SIDE acquires in each repetition time (TR) a volume that consists of interleaved slice groups, each group corresponding to a different diffusion wavevector. Undersampling occurs at the slice level when some SIDE volumes are skipped for acceleration at the expense of full spatial coverage for each wavevector. Recovering full DW images from undersampled SIDE data is inherently ill-posed. To tackle this problem, we utilize a diffusion spectrum model and total variation regularization to constrain the reconstruction of full DW images. We solve the large-scale inverse problem efficiently using the alternating direction method of multipliers (ADMM) (Yang et al., 2013; Chan et al., 2011; Chengbo, 2009) for distributed convex optimization.

We compared DW images estimated from SIDE data, acquired with a range of undersampling factors, with fully sampled data. Evaluation results covering reconstructed images, microstructural properties, and tractography streamlines indicate that our reconstruction strategy, combined with SIDE undersampling and SMS acceleration, is capable of reducing the acquisition time by a factor as much as 25, making it possible to acquire data associated with a larger number of diffusion encodings in a lesser amount of time.

## 2. Background

In this section, we provide a brief summary on spherical deconvolution and restriction spectrum imaging, which form the basis of our reconstruction algorithm.

### 2.1. Spherical Convolution

The DW signal can be represented by the spherical convolution of a response function with a fiber orientation distribution function (fODF) (Tournier et al., 2007):

(1) $$E(q)=S(q)/S_{0}=\int_{S^{2}}R(q;v)f(v)dv,$$

where **q** is the diffusion wavevector, $S^{2}$ is the unit sphere, *R*(·) is an antipodal and axial symmetric response function representing a coherent axonal bundle, *f*(·) is the fODF, *S*_0_ is the signal measured with no diffusion weighting, and *S*(**q**)/*S*_0_ is the signal attenuation. The fiber response function can be parameterized with transverse diffusivity *D*_T_ and longitudinal diffusivity *D*_L_ with *D*_L_ ≥ *D*_T_ as

(2) $$R(q;v)=\text{exp}\left(-b\left((D_{L}-D_{T})(\hat{q}\cdot v{)}^{2}+D_{T}\right)\right),$$

where *b* = ∣**q**∣^2^(Δ −*δ*/3) is the diffusion weighting, $\hat{q}=q\;∕|q|$ is a unit vector along **q**, and Δ and *δ* are respectively the time between the onsets of two gradient pulses and the pulse length in a pulsed gradient spin echo (PGSE) sequence Stejskal and Tanner (1965). The fODF is often parameterized using a set of spherical harmonics (SHs) *𝒴*:

(3) $$f(v)=\sum_{p=1}^{P}\beta_{p}{𝒴}_{p}(v),$$

where *P* is the total number of SH basis functions, and {*β*_1_, *β*_2_, …, *β*_*P*_} are real-valued weights that need to be estimated. Given *N*_*Q*_ diffusion wavevectors {**q**_1_, …, **q**_*N_Q_*_} and *N*_F_ fODF reconstruction points on the sphere {**v**_1_, …, **v**_*N*_F__}, (1) can be written in matrix form as **e** = **RY*β***, with

(4) $$e\;=\left[\begin{array}{c} E(q_{1})\\ \vdots \\ E\left(q_{{}_{N_{Q}}}\right) \end{array}\right],\;\;R=\left[\begin{array}{ccc} R(q_{1};v_{1}) & \cdots & R(q_{1};v_{N_{F}})\\ \vdots & \ddots & \vdots \\ R\left(q_{{}_{N_{Q}}};v_{1}\right) & \cdots & R(q_{Q};v_{N_{F}}) \end{array}\right],\;Y\;=\left[\begin{array}{ccc} {𝒴}_{1}(v_{1}) & \cdots & {𝒴}_{P}(v_{1})\\ \vdots & \ddots & \vdots \\ {𝒴}_{1}(v_{N_{F}}) & \cdots & {𝒴}_{P}(v_{N_{F}}) \end{array}\right],\;\;\text{and}\;\;\beta =\left[\begin{array}{c} \beta_{1}\\ \vdots \\ \beta_{P} \end{array}\right].$$

### 2.2. Diffusion Spectrum Model

We extend the spherical convolution model by using restriction spectrum imaging (RSI) (White et al., 2013), which was developed to probe the orientation structure of tissue architecture over a spectrum of length scales with minimal assumption on the underlying microstructure. The spherical convolution model assumes that the tissue architecture can be described by a linear mixture of cylindrical fiber elements with identical diffusion characteristics. In other words, the diffusion length scale is presumed fixed for all fibers within and across voxels. To do away with this assumption, RSI implements a straightforward extension by allowing for a mixture of spherical convolution models for different *D*_T_’s and a constant *D*_L_:

(5) $$e\;=\sum_{k=1}^{K}R_{k}Y_{k}\beta_{k}\;\;=\underset{A}{\underset{︸}{\left[R_{1}Y_{1}\cdots R_{K}Y_{K}\right]}}\underset{v}{\underset{︸}{\left[\begin{array}{c} {\beta_{1}}^{\vdots \;{}^{{}^{\beta_{K}}}} \end{array}\right]}},$$

where *K* is the number of distinct sets of *D*_T_ and *D*_L_. This model allows the diffusion MRI signal to be divided into parts over a range of length scales with each part associated with a response function and a fODF. Note that unlike RSI, we relax the restriction on *D*_L_ and allow it to vary.

## 3. Methods

Here, we introduce a robust method for reconstructing DW images from highly undersampled SIDE data. We first describe the SIDE acquisition strategy and then the reconstruction method.

### 3.1. SIDE Acquisition

**Fig. 1.**
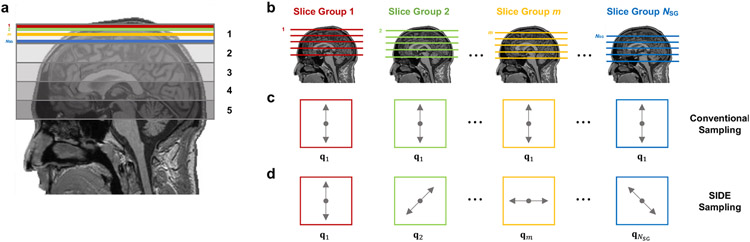
SIDE acquisition. (a) SMS acquisition example for factor 5. Each SMS excitation covers a group of slices, each in one of the 5 regions. (b) Multiple slice groups cover a volume. (c) Conventional one-volume-one-encoding scheme with SMS slice groups for each volume associated a single diffusion wavevector. (d) SIDE acquisition with SMS slice groups associated with different diffusion wavevectors.

The one-volume-one-encoding paradigm employed in conventional diffusion imaging using single-shot echo planar imaging (EPI) implies that one repetition time (TR), typically in the order of seconds, is required to cover one volume associated with one diffusion encoding. The total acquisition time in a dMRI experiment is proportional to the number of diffusion encodings that need to be covered. Reducing the TR to speed up acquisition can have undesirable consequences such as lower signal-to-noise ratio (SNR) and increased spin-history artifacts. SIDE acquires for each TR a volume consisting of interleaved slice groups, each corresponding to a different diffusion wavevector. This allows SIDE to rapidly acquire information associated with a large number of wavevectors within a short period of time at the expense of full spatial coverage for each wavevector. SIDE acquisition is illustrated in Fig. 1.

In SIDE, each simultaneous multi-slice (SMS) excitation covers a group of *R*_SMS_ slices, and *N*_SG_ slice groups (SGs) cover the whole brain (Figure 1(a)). In the conventional dMRI acquisition scheme, all SGs in a volume share the same diffusion wavevector (Figure 1(c)). In contrast, SIDE encodes each SG in a volume with a different diffusion wavevector (Figure 1(d)), e.g., **q**_1_ for SG 1, **q**_2_ for SG 2, and so on. Each SIDE volume covers *N*_SG_ wavevectors. A total of *N*_Q_ wavevectors can be covered with *N*_Q_/*N*_SG_ SIDE volumes and one SG per wavevector. *N*_Q_/*N*_SG_ SIDE volumes form a *cycle*. The wavevectors are shifted by an offset from cycle to cycle so that a different SG is covered for each wavevector. *N*_SG_ cycles cover all SGs for all wavevectors, giving a total of *N*_Q_ SIDE volumes. A subset of the cycles can be selectively acquired to achieve a certain SIDE undersampling factor *R*_SIDE_. For example, *R*_SIDE_ = 2, only half of the cycles are acquired. In combination with SMS imaging, the total undersampling factor is *R*_SMS_*R*_SIDE_.

### 3.2. Reconstruction

**Fig. 2.**
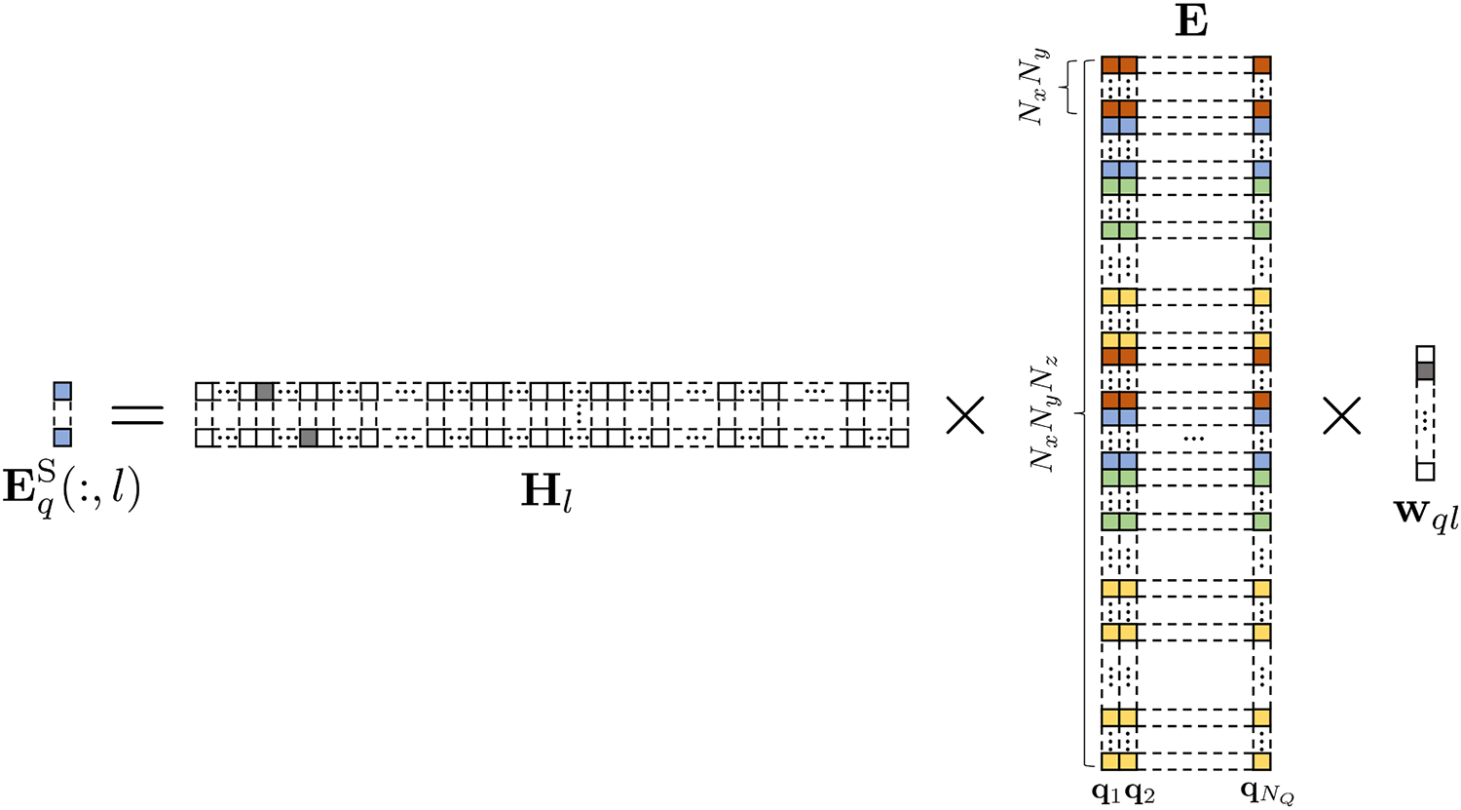
Forward model. Each column of **E** corresponds to a DW volume (*N*_*x*_ × *N*_*y*_ × *N*_*z*_) associated with a diffusion wavevector **q**. Voxels within a slice (*N*_*x*_ × *N*_*y*_) are indicated using the same color. Vector **w**_*ql*_ selects a column from **E**. Matrix **H**_*l*_ selects from **E** a number of rows that are associated with the same slice.

Using a linear forward model, the SIDE volumes can be seen as generated from fully-sampled DW volumes via subsampling operators. For convenience, we reshape the DW volumes, each with *N*_Vox_ voxels, into a matrix $E\in R^{N_{\text{Vox}}\times N_{Q}}$, where *N*_Q_ is the number of wavevectors. The SIDE volumes $\{E_{q}^{{}^{{}_{S}}}{\}}_{q=1}^{N_{Q}}$ can be represented as

(6) $$E_{q}^{{}^{{}_{S}}}(\;:,l)=H_{l}Ew_{ql}+n_{ql},$$

where $E_{q}^{{}^{{}_{S}}}(:,l)$ is the *l*-th column vector of the *q*-th SIDE volume $E_{q}^{{}^{{}_{S}}}$ and represents the *l*-th observed slice along the slice-encoding direction, **n**_*ql*_ is the measurement noise, matrix **H**_*l*_ is a slice selector along the slice-encoding direction, and vector **w**_*ql*_ is a wavevector selector. $E_{q}^{{}^{{}_{S}}}$ covers slices for multiple wavevectors. The forward model is summarized in Figure 2. For undersampling by factor *R*_SIDE_, only a subset of the SIDE volumes, i.e., *N*_Q_/*R*_SIDE_, are acquired.

The simplest means to reconstruct **E** from a subset of SIDE volumes is to solve

(7) $$\underset{E}{\text{min}}\frac{1}{2}\sum_{q,l}\|\;H_{l}Ew_{ql}-E_{q}^{{}^{{}_{S}}}(\;:,l)\;{\|}_{{}_{F}}^{{}^{2}},$$

where ∥ · ∥_F_ is the Frobenius norm. However, the inverse problem is ill-posed and needs to be regularized, as discussed next.

### 3.3. Regularization

Problem (7) can be spatially regularized using the total variation (TV) semi-norm. Denoting **E**^(*q*)^ as the *q*-th column of **E** reshaped to a *N*_*x*_ ×*N*_*y*_ × *N*_*z*_ tensor, the TV semi-norm of **E**^(*q*)^ is

(8) ‖E(q)‖TV=∑i=1Nx∑j=1Ny∑k=1Nz(γx2[Eijk(q)−E(i+1)jk(q)]2+γy2[Eijk(q)−Ei(j+1)k(q)]2)+(γz2[Eijk(q)−Eij(k+1)(q)]2)12,

where *γ*_*x*_, *γ*_*y*_, and *γ*_*z*_ are tuning parameters that control the regularization in the different spatial dimensions. The TV of **E** is defined as

(9) $$\|\;E\;{\|}_{\text{TV}}=\sum_{q=1}^{N_{Q}}\|\;E^{\left(q\right)}\;{\|}_{\text{TV}}.$$

Based on (5) and letting **V** = [**v**_1_, …, **v**_*N*_Vox__], we can constrain **E** by letting **E**^⊤^ = **AV**. A regularized version of (7) can be written as

(10) $$\underset{E}{\text{min}}\;\;\frac{1}{2}\sum_{q,l}\|\;H_{l}Ew_{ql}-E_{q}^{{}^{{}_{S}}}(\;:,l)\;{\|}_{{}_{F}}^{{}^{2}}+\lambda \|\;E\;{\|}_{\text{TV}}\;\;\text{s.t.}\;\;E^{T}=\mathrm{AV},$$

where parameter *λ* is non-negative and controls the tradeoff between data fidelity and regularization.

### 3.4. Optimization

We utilize the alternating direction method of multipliers (ADMM) (Yang et al., 2013; Chan et al., 2011; Chengbo, 2009) to solve (10) by minimizing the following function:

(11) $$L(E,V,\;\;\{U_{q}{\}}_{q=1}^{N_{Q}},\{\Psi_{q}{\}}_{q=1}^{N_{Q}},\Phi )=\;\;\frac{1}{2}\sum_{q,l}\|\;H_{l}Ew_{ql}-E_{q}^{{}^{{}_{S}}}(\;:,l)\;{\|}_{{}_{F}}^{{}^{2}}+\lambda \sum_{q=1}^{N_{Q}}\|\;U_{q}{\|}_{\text{TV}}+\;\;\frac{\rho_{1}}{2}\sum_{q=1}^{N_{Q}}\|\;U_{q}-E^{\left(v\right)}+\Psi_{q}\;{\|}_{{}_{F}}^{{}^{2}}+\frac{\rho_{2}}{2}\|\;E^{T}-\mathrm{AV}+\Phi \;{\|}_{{}_{F}}^{{}^{2}}.$$

We carry out the following steps to minimize the cost function $L(E,V,\{U_{q}{\}}_{q=1}^{N_{Q}},\{\Psi_{q}{\}}_{q=1}^{N_{Q}},\Phi )$ over **E, V**, and auxiliary variables $\{U_{q}{\}}_{q=1}^{N_{Q}}$, and update Lagrangian multipliers $\{\Psi_{q}{\}}_{q=1}^{N_{Q}}$ and **Φ** in each iteration *t*:

1. **Subproblem 1** - Update **V** by minimizing
(12) $$V(t+1)=\underset{V}{\text{argmin}}\;\frac{\rho_{2}}{2}\|\;E^{T}(t)-\mathrm{AV}+\Phi (t)\;{\|}_{2}^{2}.$$
2. **Subproblem 2** - Update $\{U_{q}{\}}_{q=1}^{N_{Q}}$ by minimizing
(13) $$U_{q}(t+1)\;=\underset{U_{q}}{\text{argmin}}\;\frac{\rho_{1}}{2}\sum_{q=1}^{N_{Q}}\|\;U_{q}-E^{\left(q\right)}(t)+\Psi_{q}(t)\;{\|}_{2}^{2}\;\;+\lambda \sum_{q=1}^{N_{Q}}\|\;U_{q}\;{\|}_{\text{TV}}$$
  via the efficient algorithm described in (Chan et al., 2011).
3. **Subproblem 3** - Update **E** by minimizing
(14) $$E(t+1)=\;\underset{E}{\text{argmin}}\;\frac{1}{2}\sum_{q,l}\|\;H_{l}Ew_{ql}-E_{q}^{{}^{{}_{S}}}(\;:,l)\;{\|}_{{}_{F}}^{{}^{2}}+\;\;\frac{\rho_{1}}{2}\sum_{q=1}^{N_{Q}}\|\;U_{q}(t+1)-E^{\left(q\right)}+\Psi_{q}(t)\;{\|}_{{}_{F}}^{{}^{2}}\;\;+\frac{\rho_{2}}{2}\|\;E^{T}-\mathrm{AV}(t+1)+\Phi (t)\;{\|}_{{}_{F}}^{{}^{2}}.$$
4. **Subproblem 4** - Update ***Ψ*** and **Φ** by
(15) $$\Psi_{q}(t+1)=\Psi_{q}(t)+\left(U_{q}(t+1)-E^{\left(q\right)}(t+1)\right)$$
  and
(16) $$\Phi (t+1)=\Phi (t)+(E^{T}(t+1)-\mathrm{AV}(t+1))$$
  A summary is provided in Algorithm 1.

## 4. Experiments

### 4.1. Materials

SIDE diffusion MRI data from seven healthy subjects were acquired using a 3T Siemens scanner (Siemens Healthcare, Erlangen, Germany). Diffusion imaging was performed with a monopolar diffusion weighted PGSE-EPI sequence. SMS RF excitation with controlled aliasing (blip-ped-CAIPI) was employed to reduce the penalty of geometry factor (g-factor). Imaging parameters were as follows: Resolution = (1.5 mm)^3^; FOV = 192 × 192 × 150 mm^3^; image dimensions = 128 × 128 × 100; partial Fourier = 6/8; SMS factor *R*_SMS_ = 5; bandwidth = 1776 Hz /Px; 160 wavevectors distributed over the 4 b-shells of *b* = 500, 1000, 2000, and 3000 s/mm^2^ with 16, 32, 48, and 64 non-collinear directions, respectively, plus one *b* = 0 s/mm^2^ scan; TR /TE = 3120/90 ms; and 32-channel head array coil. The total acquisition time is 8 min 19 s for each phase-encoding direction (anterior-posterior or posterior-anterior). Note that *N*_Q_ = 160 and *N*_SG_ = 100/5 = 20.

Diffusion MRI data of a pediatric subject, scanned at 146 days, 223 days, and 423 days after birth via the Baby Connectome Project (BCP) (Howell et al., 2019), were retrospectively undersampled to mimic SIDE acquisition using a factor of 4. The data were collected with 1.5 mm isotropic resolution with *b* = 500, 1000, 1500, 2000, 2500, 3000 s/mm^2^ using a total of 144 gradient directions, multi-band factor 5, and positive and negative phase encoding in the anterior-posterior direction. Six non-diffusion-weighted images were also acquired. The total scan time was approximately 13 min.

### 4.2. Results

We compared SIDE undersampling with conventional undersampling via reducing the number of wavevectors. For each SIDE subsampling factor, conventional undersampling reduces the acquisition time by reducing the angular samples. In contrast, SIDE subsampling retains the angular resolution but reduces the number of sampled slices (see Table 1). The number of acquired volumes was kept the same for both cases to keep the acquisition time identical. SIDE acquisition covers a larger number of wavevectors with the same number of volumes. For fair comparison, the DW images at full angular resolution were recovered using our reconstruction algorithm for both SIDE and conventional undersampling.

To cover the diffusion spectrum, we set the isotropic diffusivity to [0, 3] × 10^−3^ mm^2^/s, the longitudinal diffusivity *D*_*L*_ to [1.5, 2.5] × 10^−3^ mm^2^/s, and the radial diffusivity *D*_T_ according to *D*_L_/*D*_T_ ≥ 1.1 as in (Huynh et al., 2020; 2019). We set (*γ*_*x*_, *γ*_*y*_, *γ*_*z*_) = (0.9, 0.9, 1), (*ρ*_1_, *ρ*_2_) = (0.5, 0.5), and *λ* = 0.01. Spherical harmonics up to order eight were used for fODF representation. Reconstruction accuracy was quantified using the following metrics:

1. Normalized mean squared error (NMSE) between the estimation **E** and ground truth $\tilde{E}$, defined as $\|\;E-\tilde{E}{\|}_{F}∕N_{\text{Vox}}$.
2. Peak signal-to-noise ratio (PSNR) is defined as $10\log_{10}\frac{\text{MAX}}{\text{NMSE}}$, where MAX is the maximum signal value.
3. Structural similarity index (SSIM) as defined in (Wang et al., 2004).
4. Relative difference defined as ∣*x* − *x*_GT_∣/*x*_GT_, where *x* and *x*_GT_ are respectively the predicted and ground-truth values.
5. Fiber bundle overlap (FBO) defined as 1 − ∑_*i*_∣*p*(*i*) − *p*_GT_(*i*)∣/∑_*i*_*p*_GT_(*i*), where *p*(*i*) and *p*_GT_(*i*) are the respective probabilities that the *i*-th voxel is part of the predicted and ground-truth fiber bundles.

The following microstructural scalars were considered:

1. Generalized fractional anistropy (GFA) as defined in (Tuch, 2004; Yamada et al., 2018).
2. Orientation dispersion (OD), neurite density (ND), and free water (FW) based on NODDI (Zhang et al., 2012) and computed via AMICO (Daducci et al., 2015).

**Fig. 3.**
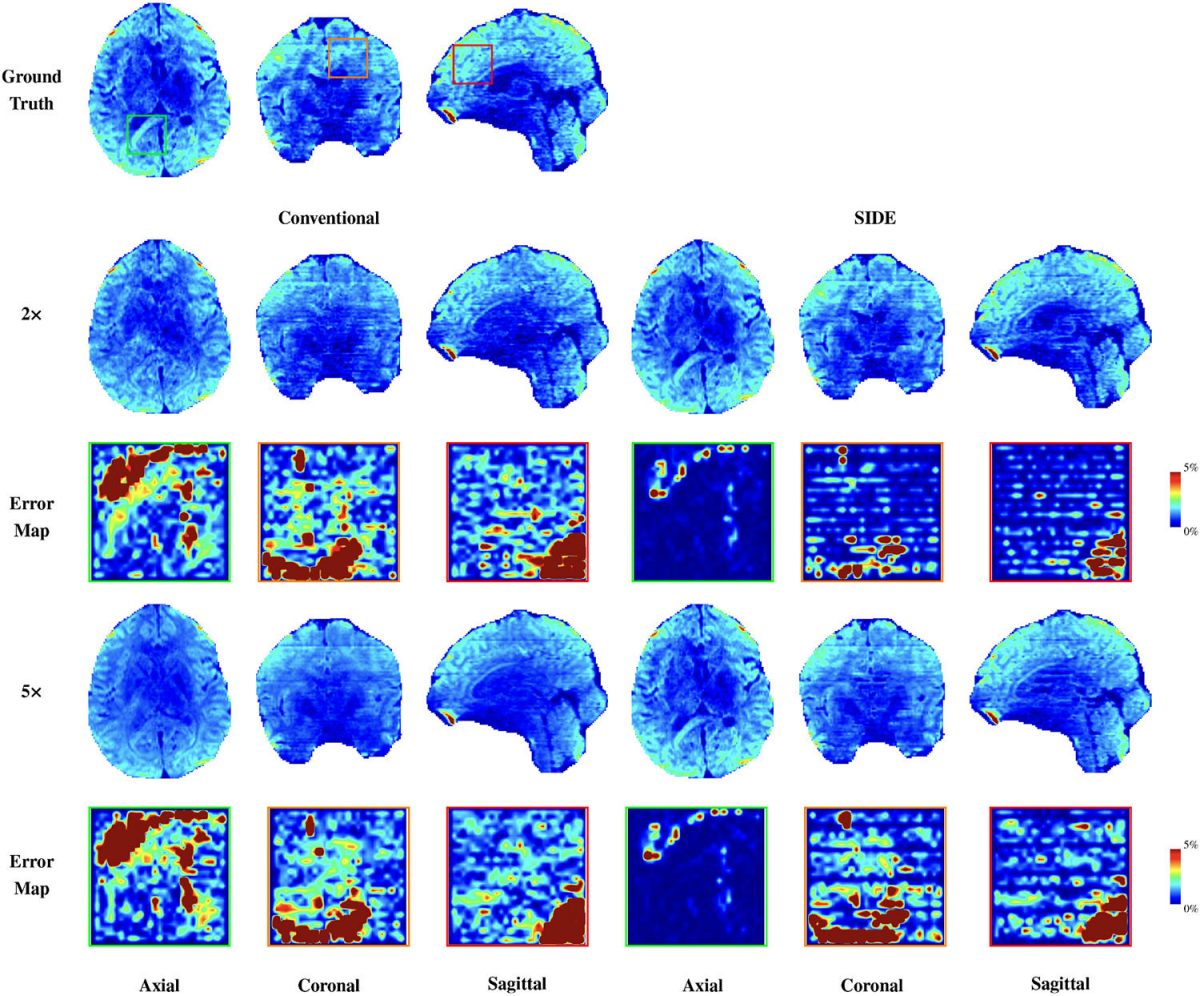
DW images (*b* = 2000 s/mm^2^) reconstructed from SIDE and conventional undersampling data for factors 2 and 5. The corresponding error maps, computed based on the relative difference, are shown for selected regions.

Figure 3 shows the axial, coronal, and sagittal views of a DW volume from the fully-sampled data and the reconstructed data based on SIDE and conventional undersampling for two undersampling factors 2 and 5. It is clear that, when compared with SIDE sampling, conventional sampling is not effective for recovering the details in DW images. This observation is confirmed quantitatively by the error maps, indicating an average error of about 2% for gray and white matter for SIDE sampling, which is significantly lower than conventional sampling.

**Fig. 4.**
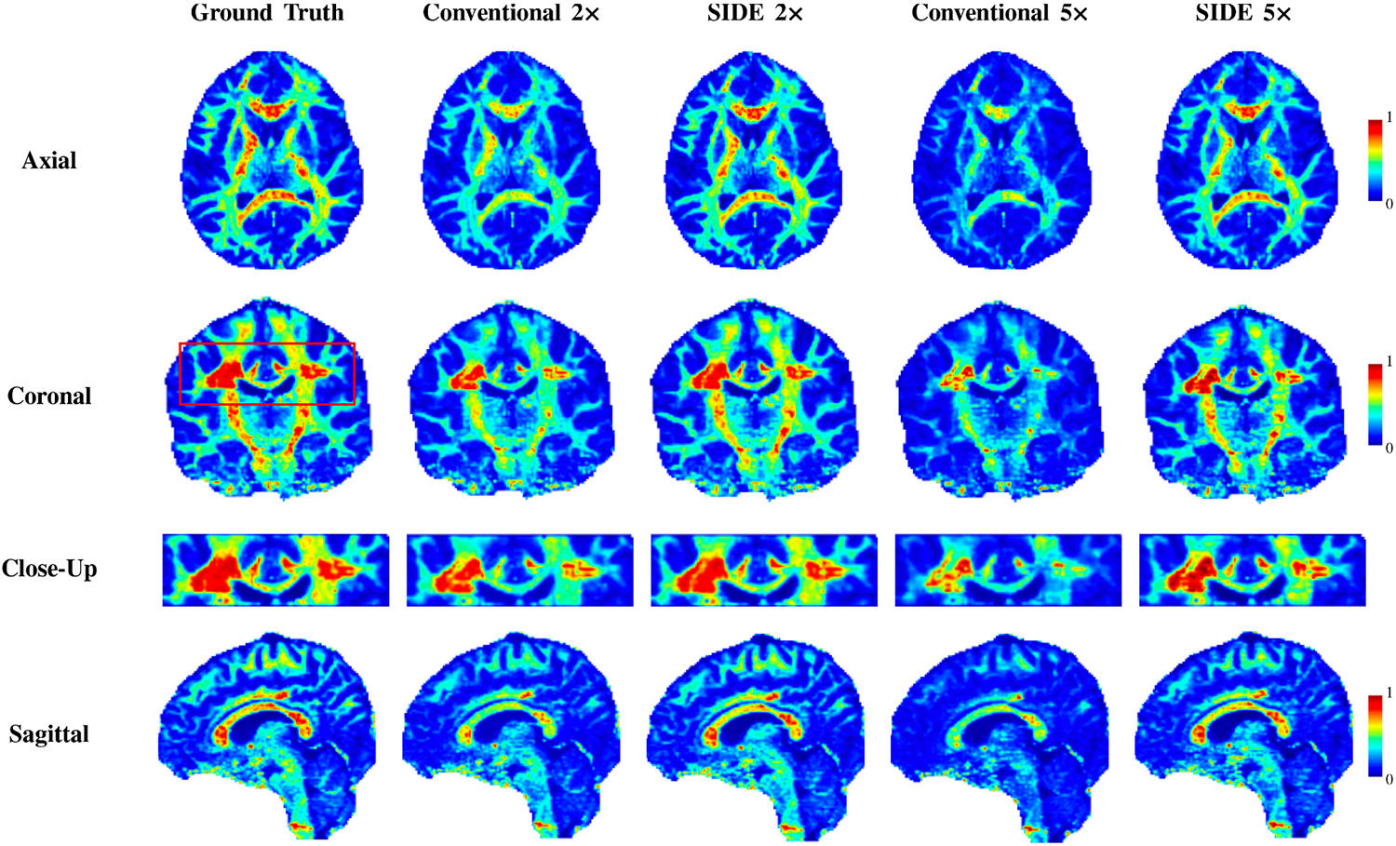
GFA maps of DW images estimated from fully-sampled, SIDE, and conventional undersampling data for undersampling factors 2 and 5.

Figure 4 shows that SIDE sampling yields significantly higher generalized fractional anisotropy (GFA) values than conventional sampling in white matter (see the corpus callosum). This indicates that SIDE sampling is less prone to sacrificing angular resolution. Quantitative results for reconstructed DW images and the corresponding GFA maps are shown in Table 2, comparing reconstruction performance with single-shell and multi-shell acquisitions for different undersampling factors. Both the DW image and GFA results indicate that reconstruction is most effective when multiple shells are considered jointly. As expected, as the undersampling factor increases, the performance degrades. However, for high undersampling factors, more accurate reconstruction can be achieved with SIDE sampling.

**Fig. 5.**
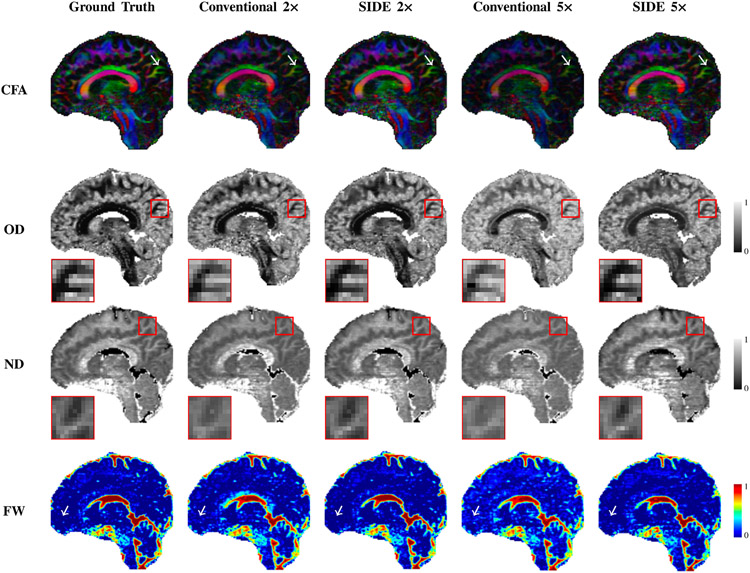
Colored fractional anisotropy (CFA), orientation dispersion (OD), neurite density (ND), and free water (FW) maps of diffusion-weighted volumes estimated from fully-sampled, SIDE, and conventional undersampling data for factors 2 and 5.

Figure 5 shows that, when the undersampling factor is high, conventional sampling causes the over-estimation of neurite density (ND) and orientation dispersion (OD), computed based on NODDI (Zhang et al., 2012). Quantitative results for neurite density, orientation dispersion, and free water are shown in Figure 7 for various undersampling factors. NMSE monotonically increases and PSNR and SSMI monotonically decrease with the undersampling factor. The results again confirm that joint consideration of information from multiple shells improves reconstruction.

Figure 8 shows the fODFs in regions with crossing, bifurcating, and coherent fibers. In the region with crossing fibers (first row of Figure 8), SIDE yields similar fiber orientations as the ground truth and has less spurious peaks than conventional sampling. In the region with coherent fibers (second row of Figure 8), SIDE yields smoother fiber orientations than the conventional scheme. In the region with bifurcating fibers (third row in Figure 8), SIDE resolves small angles better than the conventional scheme. The results indicate that SIDE reconstruction gives clean and coherent fODFs that resemble the ground truth. In contrast, conventional sampling produces fODFs with a large number of spurious peaks.

We performed deteministic whole-brain tractography based on spherical deconvolution (SD) (Tournier et al., 2012) using the fully-sampled and undersampled SIDE data and extracted the cingulum bundle (CB), corticospinal tract (CST), and a part of the corpus callosum (CC) bundle (Figure 9). The results indicate significant resemblance between the streamlines estimated from the SIDE data with the ground truth, despite a high undersampling factor of 5. We computed the average relative difference of the fractional anisotropy (FA), ND, and OD values of voxels in masks defined by the ground-truth fiber bundles. The results, shown in Table 3, indicate that SIDE yields a more significant overlap between the fiber streamlines.

**Fig. 6.**
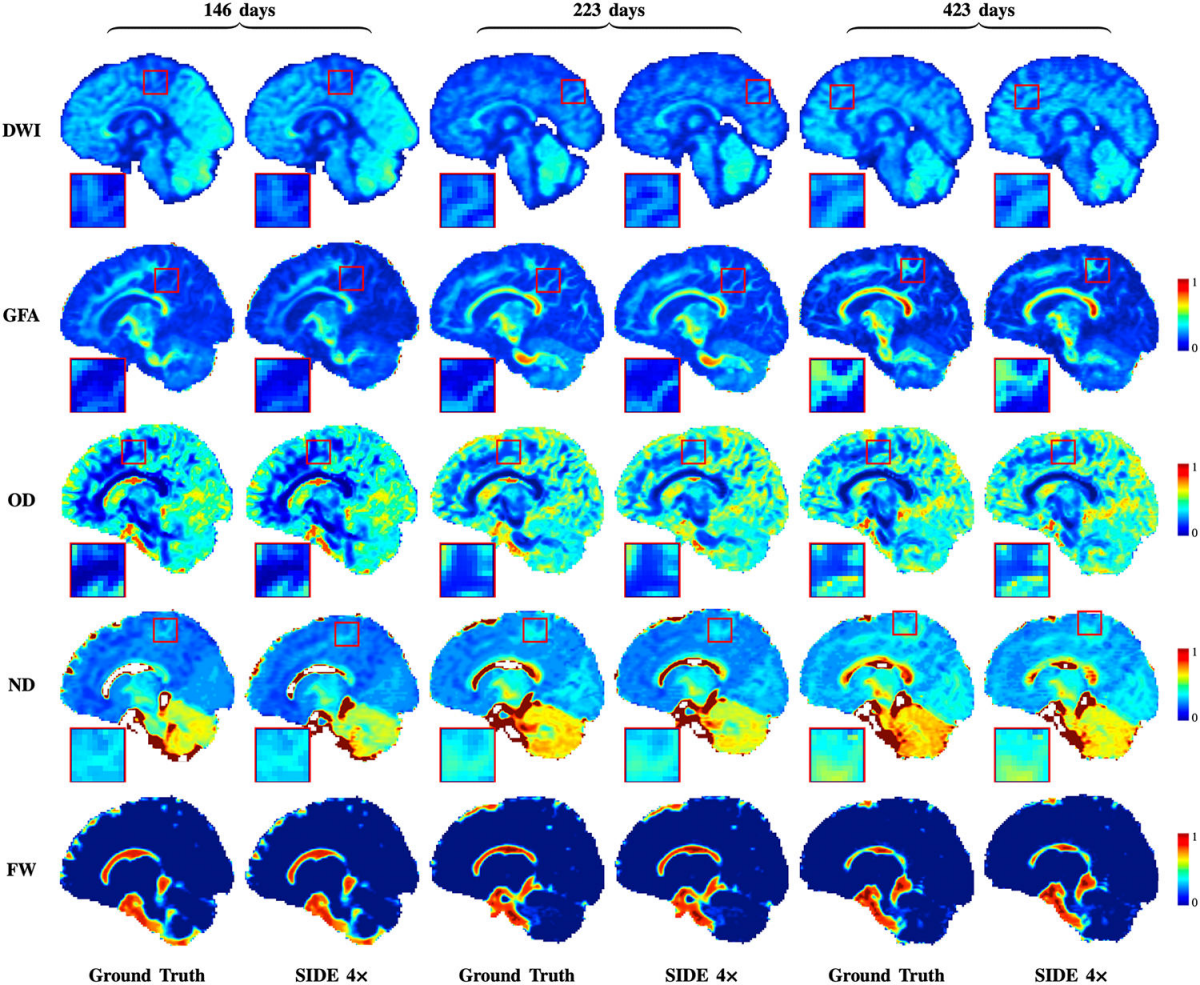
Reconstruction from retrospectively undersampled data (factor 4) of a pediatric subject scanned at 146 days, 223 days, and 423 days after birth.

**Fig. 7.**
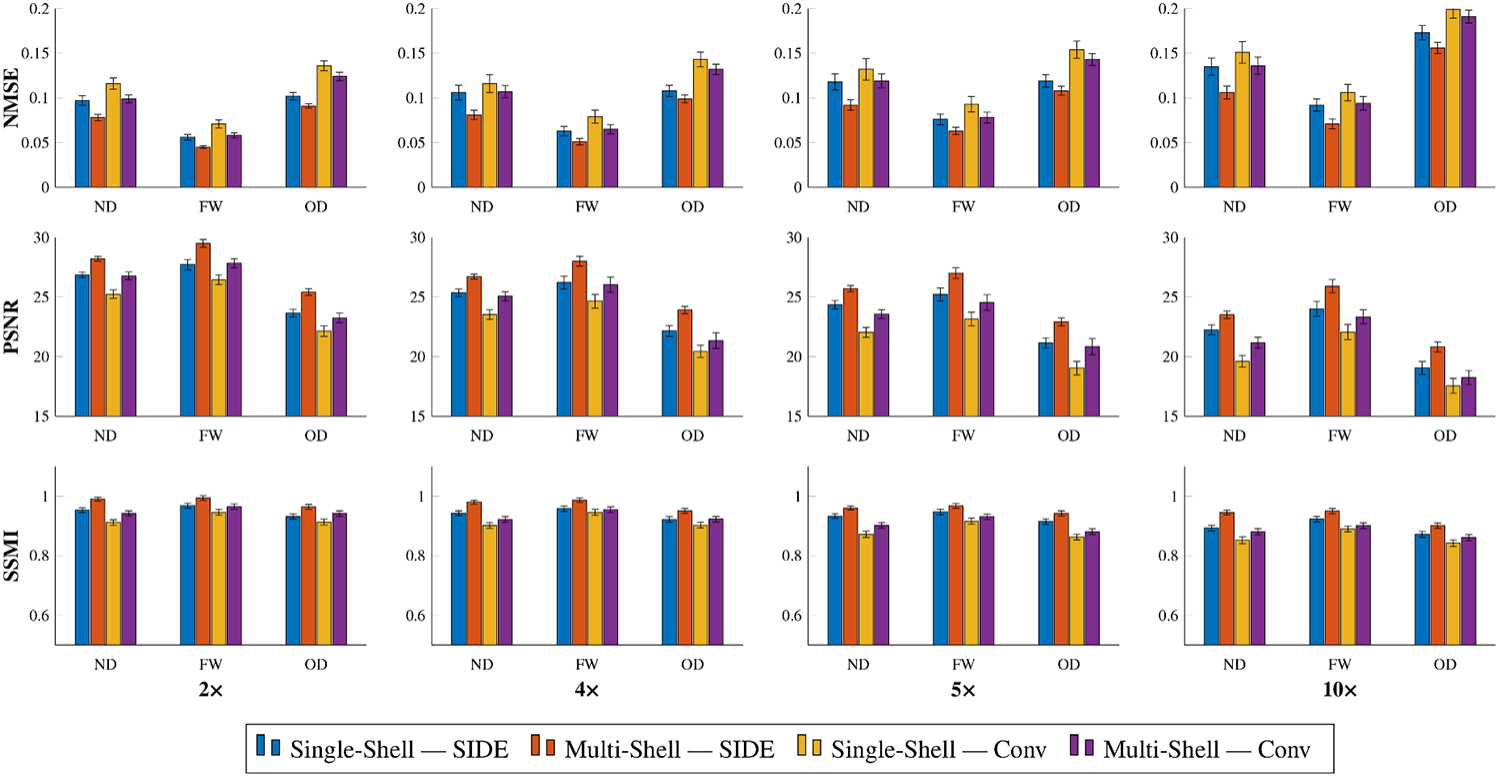
Quantitative validation based on neurite density (ND), free water (FW), and orientation dispersion (OD) for different undersampling factors.

**Fig. 8.**
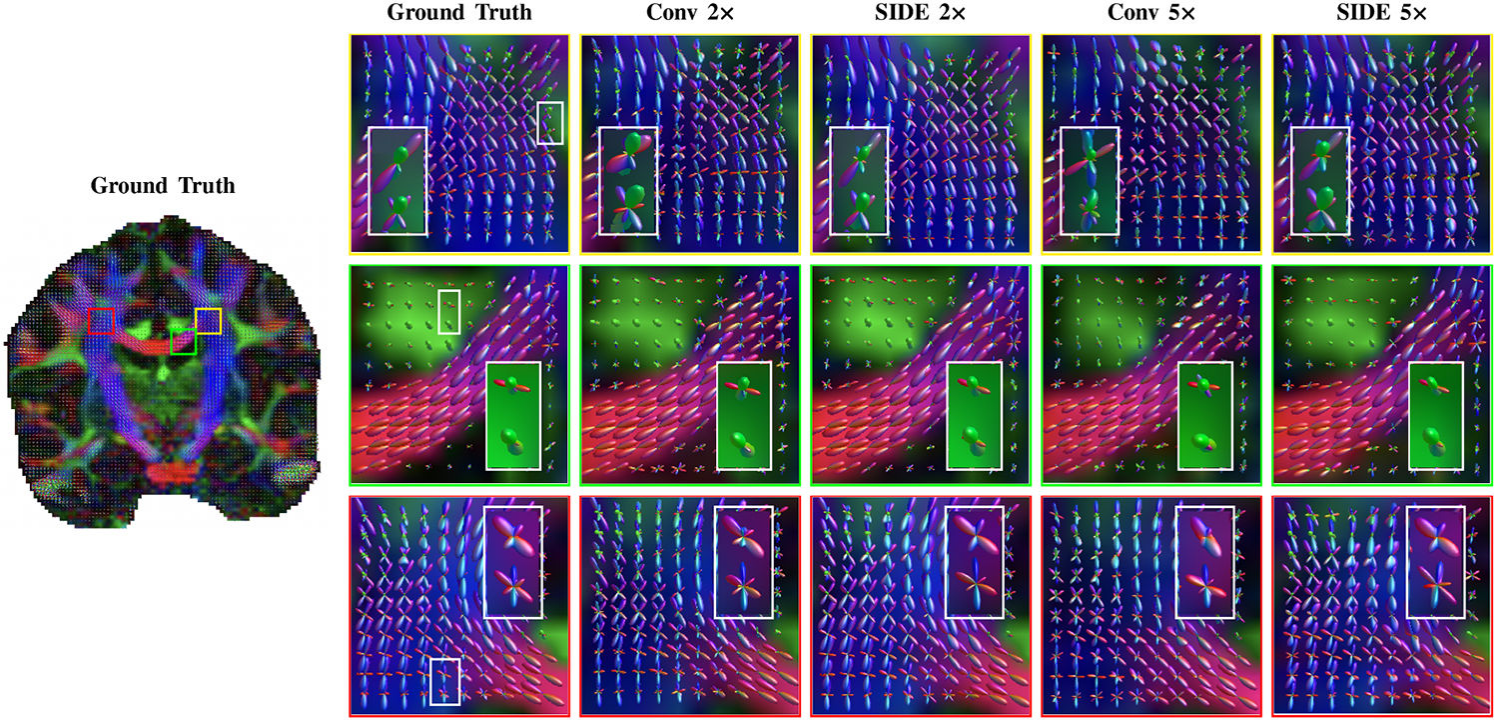
Fiber ODFs in directionally-encoded colors (red: left-right, green: anterior-posterior, blue: inferior-superior) in regions with crossing, bifurcating, and unidirectional fibers, generated from SIDE and conventional undersampling data for factors 2 and 5.

**Fig. 9.**
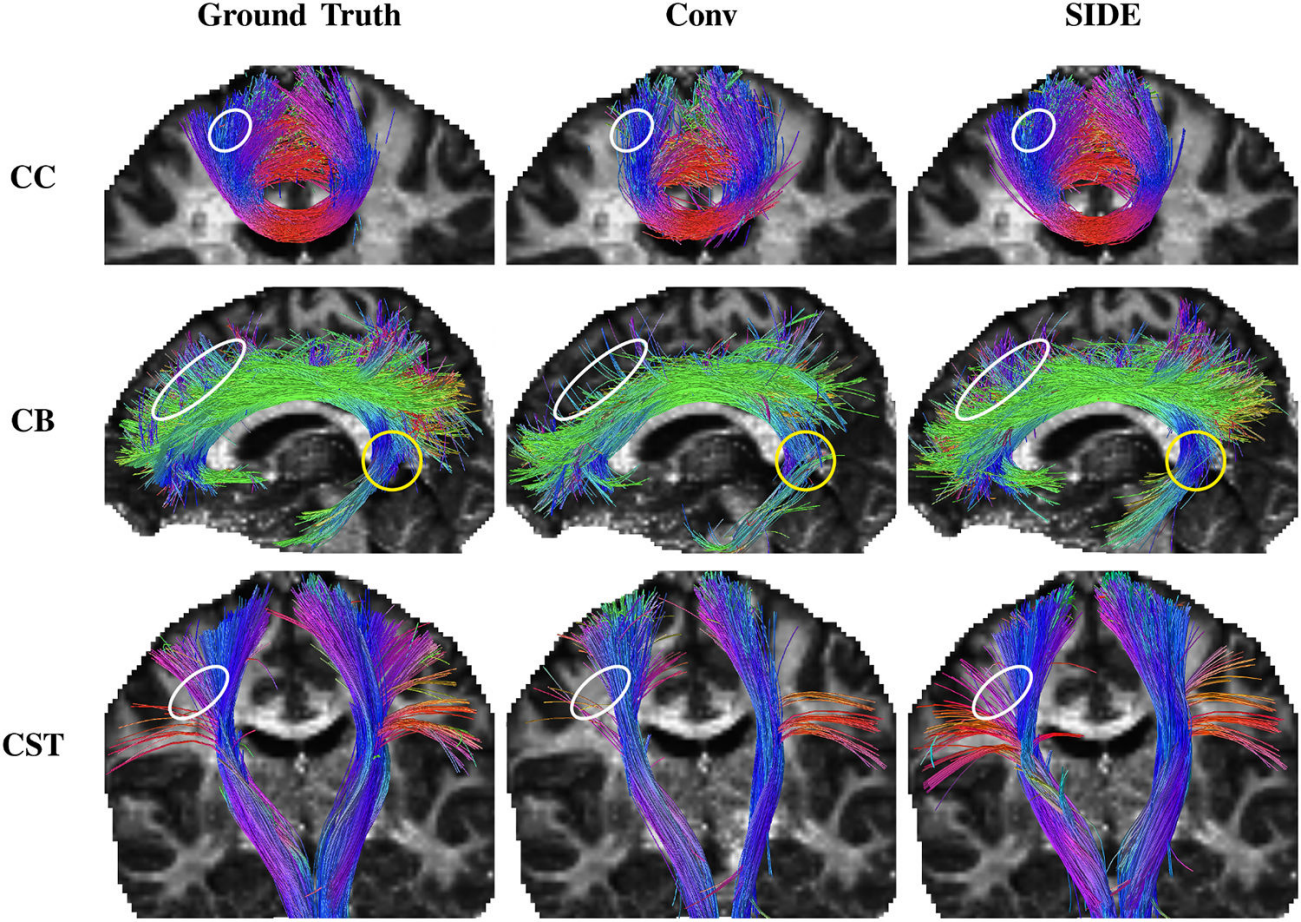
The fiber tracts, color-coded based on local tract orientations (red: left-right, green: anterior-posterior, blue: inferior-superior), of the corpus callosum (CC), cingulum bundle (CB), and corticospinal tract (CST) generated from data undersampled by a factor of 5. The ellipses mark some missing and incorrect fiber streamlines given by conventional sampling compared with the ground truth. The yellow circles mark prematurely terminated fiber streamlines given by conventional sampling.

Additional results based on the pediatric data are shown in Figure 6. The reconstructed DW images and the corresponding microstructure maps indicate that the proposed method yields results that are very similar to the ground truth.

## 5. Discussion and Conclusion

In this work, we proposed a reconstruction scheme for diffusion MRI data acquired using SIDE, realized using a diffusion spectrum model, total variation regularization, and ADMM optimization. We demonstrated that, for each DW volume, only a subsample of slices are needed to reconstruct the full 3D volumes with high fidelity even for high undersampling factors. Compared with conventional sampling, SIDE collects data with higher incoherence in the same amount time and hence results in dramatically improved reconstruction quality.

Conventional dMRI acquisition typically covers the slices associated with each diffusion gradient fully before proceeding with the next gradient. In contrast, SIDE acquisition covers the slice groups of all gradients in each cycle and repeats in the next cycle with a gradient offset. In other words, each cycle covers partial information of all gradients. This is the key characteristic of SIDE that allows unsampled information to be recovered from the data acquired in a few cycles.

Post-acquisition motion-correction algorithms may benefit from SIDE acquisition, for example, by registering motion-affected data to motion-free data. If SIDE acquisition is able to complete motion-free for a few cycles, the acquired data, which cover all gradients, can be used as a reference for registration-based motion correction. A reference for registration-based correction can be constructed by generating the full data from the motion-free data using our reconstruction framework.

SIDE dMRI will improve success in imaging motion-prone individuals such as children, elderly, and patients with conditions. It will also open up the potential for acquiring data with ultra-dense coverage of q-space with potentially thousands of sampling points, promoting the development of methods for a more complete picture of tissue microstructure using models with complexity that is less constrained by the number of data points available for model fitting.

## Figures and Tables

**Table 1:** Number of gradient directions for each diffusion weighting (s/mm^2^) given by conventional/SIDE sampling for each acceleration factor.

Conv / **SIDE**	500	1000	2000	3000	Total
1 ×	16 / **16**	32 / **32**	48 / **48**	64 / **64**	160 / **160**
2 ×	8 / **16**	16 / **32**	24 / **48**	32 / **64**	80 / **160**
5 ×	3 / **16**	6 / **32**	9 / **48**	14 / **64**	32 / **160**
10 ×	2 / **16**	3 / **32**	5 / **48**	6 / **64**	16 / **160**

**Table 2:** Numerical results for the reconstructed DW images and the corresponding GFA maps.

		2 ×		4 ×		5 ×		10 ×	
Conv / SIDE		Single-Shell	Multi-Shell	Single-Shell	Multi-Shell	Single-Shell	Multi-Shell	Single-Shell	Multi-Shell
DWI	NMSE	0.176 / **0.124**	0.153 / **0.097**	0.191 / **0.131**	0.164 / **0.105**	0.213 / **0.145**	0.169 / **0.116**	0.264 / **0.221**	0.234 / **0.189**
	PSNR	25.17 / **27.10**	26.43 / **28.27**	24.51 / **26.84**	25.44 / **28.11**	24.16 / **26.56**	24.64 / **27.65**	22.46 / **24.13**	23.56 / **25.05**
	SSIM	0.933 / **0.961**	0.945 / **0.964**	0.931 / **0.949**	0.944 / **0.953**	0.925 / **0.945**	0.939 / **0.950**	0.915 / **0.924**	0.921 / **0.931**
GFA	NMSE	0.134 / **0.095**	0.092 / **0.063**	0.153 / **0.115**	0.124 /**0.075**	0.168 / **0.130**	0.139 / **0.081**	0.204 / **0.162**	0.194 / **0.134**
	PSNR	26.89 / **28.21**	28.15 / **29.57**	25.64 / **27.95**	27.65 / **29.14**	25.13 / **27.34**	27.14 / **28.75**	23.98 / **26.01**	24.76 / **27.56**
	SSIM	0.954 / **0.971**	0.959 / **0.978**	0.948 / **0.964**	0.955 / **0.971**	0.944 / **0.958**	0.949 / **0.965**	0.921 / **0.935**	0.933 / **0.941**

**Table 3:** Numerical evaluation of fiber streamlines.

	ΔFA		ΔND		ΔOD		FBO	
	Conv	SIDE	Conv	SIDE	Conv	SIDE	Conv	SIDE
CC	3.6%	3.1%	6.5%	4.7%	7.2%	5.1%	0.92	0.98
CB	3.3%	2.8%	5.8%	4.2%	6.9%	5.4%	0.91	0.97
CST	4.6%	4.0%	7.4%	5.6%	8.7%	6.5%	0.87	0.94

## References

[R1] Alexander AL, Lee JE, Lazar M, Field AS, 2007. Diffusion tensor imaging of the brain. Neurotherapeutics 4 (3), 316–329. 17599699 10.1016/j.nurt.2007.05.011 PMC2041910

[R2] Barth M, Breuer F, Koopmans PJ, Norris DG, Poser BA, 2016. Simultaneous multislice (SMS) imaging techniques. Magn. Reson. Med 75 (1), 63–81. 26308571 10.1002/mrm.25897 PMC4915494

[R3] Basser PJ, Mattiello J, LeBihan D, 1994. Estimation of the effective self-diffusion tensor from the NMR spin echo. J. Magn. Reson. B 103 (3), 247–254. 8019776 10.1006/jmrb.1994.1037

[R4] Basser PJ, Pierpaoli C, 2011. Microstructural and physiological features of tissues elucidated by quantitative-diffusion-tensor MRI. J. Magn. Reson 213 (2), 560–570. 22152371 10.1016/j.jmr.2011.09.022

[R5] Chan SH, Khoshabeh R, Gibson KB, Gill PE, Nguyen TQ, 2011. An augmented Lagrangian method for total variation video restoration. IEEE Trans. Image Process 20 (11), 3097–3111. 21632302 10.1109/TIP.2011.2158229

[R6] Chengbo L, 2009. An efficient algorithm for total variation regularization with applications to the single pixel camera and compressive sensing. Houston: Department of Computational and Applied Mathematics.

[R7] Christiaens D, Cordero-Grande L, Pietsch M, Hutter J, Price AN, Hughes EJ, Vecchiato K, Deprez M, Edwards AD, Hajnal JV, , 2021. Scattered slice SHARD reconstruction for motion correction in multi-shell diffusion MRI. Neuroimage 225, 117437. 33068713 10.1016/j.neuroimage.2020.117437 PMC7779423

[R8] Colgan N, Siow B, O’Callaghan JM, Harrison IF, Wells JA, Holmes HE, Ismail O, Richardson S, Alexander DC, Collins EC, , 2016. Application of neurite orientation dispersion and density imaging (NODDI) to a tau pathology model of alzheimer’s disease. Neuroimage 125, 739–744. 26505297 10.1016/j.neuroimage.2015.10.043 PMC4692518

[R9] Daducci A, Canales-Rodríguez EJ, Zhang H, Dyrby TB, Alexander DC, Thiran J-P, 2015. Accelerated microstructure imaging via convex optimization (AMICO) from diffusion MRI data. Neuroimage 105, 32–44. 25462697 10.1016/j.neuroimage.2014.10.026

[R10] Ennis DB, Kindlmann G, 2006. Orthogonal tensor invariants and the analysis of diffusion tensor magnetic resonance images. Magn. Reson. Med 55, 136–146. 16342267 10.1002/mrm.20741

[R11] Hong Y, Chen G, Yap P-T, Shen D, 2019. Reconstructing high-quality diffusion MRI data from orthogonal slice-undersampled data using graph convolutional neural networks. International Conference on Medical Image Computing and Computer-Assisted Intervention. Springer, pp. 529–537. 10.1007/978-3-030-32248-9_59 PMC7065676 32161931

[R12] Howell BR, Styner MA, Gao W, Yap P-T, Wang L, Baluyot K, Yacoub E, Chen G, Potts T, Salzwedel A, , 2019. The UNC/UMN baby connectome project (BCP): an overview of the study design and protocol development. Neuroimage 185, 891–905. 29578031 10.1016/j.neuroimage.2018.03.049 PMC6151176

[R13] Huynh KM, Xu T, Wu Y, Chen G, Thung K-H, Wu H, Lin W, Shen D, Yap P-T, Consortium UBCP, , 2019. Probing brain micro-architecture by orientation distribution invariant identification of diffusion compartments. International Conference on Medical Image Computing and Computer-Assisted Intervention. Springer, pp. 547–555. 10.1007/978-3-030-32248-9_61 PMC8386182 34447975

[R14] Huynh KM, Xu T, Wu Y, Wang X, Chen G, Wu H, Thung K-H, Lin W, Shen D, Yap P-T, 2020. Probing tissue microarchitecture of the baby brain via spherical mean spectrum imaging. IEEE Trans. Med. Imaging 39 (11), 3607–3618. 32746109 10.1109/TMI.2020.3001175 PMC7688284

[R15] Jeurissen B, Tournier J-D, Dhollander T, Connelly A, Sijbers J, 2014. Multi-tissue constrained spherical deconvolution for improved analysis of multi-shell diffusion MRI data. Neuroimage 103, 411–426. 25109526 10.1016/j.neuroimage.2014.07.061

[R16] Ning L, Setsompop K, Michailovich O, Makris N, Shenton ME, Westin C-F, Rathi Y, 2016. A joint compressed-sensing and super-resolution approach for very high resolution diffusion imaging. NeuroImage 125, 386–400. 26505296 10.1016/j.neuroimage.2015.10.061 PMC4691422

[R17] Ramos-Llordén G, Ning L, Liao C, Mukhometzianov R, Michailovich O, Setsompop K, Rathi Y, 2020. High-fidelity, accelerated whole-brain submillimeter in vivo diffusion MRI using gSlider-spherical ridgelets (gSlider-SR). Magn. Reson. Med 84 (4), 1781–1795. 32125020 10.1002/mrm.28232 PMC9149785

[R18] Roine T, Jeurissen B, Perrone D, Aelterman J, Philips W, Leemans A, Sijbers J, 2015. Informed constrained spherical deconvolution (iCSD). Med. Image Anal 24 (1), 269–281. 25660002 10.1016/j.media.2015.01.001

[R19] Schneider T, Brownlee W, Zhang H, Ciccarelli O, Miller DH, Wheeler-Kingshott CG, 2017. Sensitivity of multi-shell NODDI to multiple sclerosis white matter changes: a pilot study. Funct. Neurol 32 (2), 97. 28676143 10.11138/FNeur/2017.32.2.097 PMC5507159

[R20] Shenton ME, Hamoda H, Schneiderman J, Bouix S, Pasternak O, Rathi Y, Vu M-A, Purohit MP, Helmer K, Koerte I, , 2012. A review of magnetic resonance imaging and diffusion tensor imaging findings in mild traumatic brain injury. Brain Imaging Behav. 6 (2), 137–192. 22438191 10.1007/s11682-012-9156-5 PMC3803157

[R21] Stejskal E, Tanner J, 1965. Spin diffusion measurements: spin echoes in the presence of time-dependent field gradient. J. Chem. Phys 42 (1), 288–292.

[R22] Sui J, Pearlson GD, Du Y, Yu Q, Jones TR, Chen J, Jiang T, Bustillo J, Calhoun VD, 2015. In search of multimodal neuroimaging biomarkers of cognitive deficits in schizophrenia. Biol. Psychiatry 78 (11), 794–804. 25847180 10.1016/j.biopsych.2015.02.017 PMC4547923

[R23] Tournier J-D, Calamante F, Connelly A, 2007. Robust determination of the fibre orientation distribution in diffusion MRI: non-negativity constrained super-resolved spherical deconvolution. NeuroImage 35 (4), 1459–1472. 17379540 10.1016/j.neuroimage.2007.02.016

[R24] Tournier J-D, Calamante F, Connelly A, 2012. Mrtrix: diffusion tractography in crossing fiber regions. Int. J. Imaging Syst. Technol 22 (1), 53–66.

[R25] Tuch DS, 2004. Q-ball imaging. Magn. Reson. Med 52 (6), 1358–1372. 15562495 10.1002/mrm.20279

[R26] Tuch DS, Reese TG, Wiegell MR, Makris N, Belliveau JW, Wedeen VJ, 2002. High angular resolution diffusion imaging reveals intravoxel white matter fiber heterogeneity. Magn. Reson. Med 48 (4), 577–582. 12353272 10.1002/mrm.10268

[R27] Tuch DS, Reese TG, Wiegell MR, Wedeen VJ, 2003. Diffusion MRI of complex neural architecture. Neuron 40 (5), 885–895. 14659088 10.1016/s0896-6273(03)00758-x

[R28] Vos SB, Jones DK, Viergever MA, Leemans A, 2011. Partial volume effect as a hidden covariate in DTI analyses. NeuroImage 55 (4), 1566–1576. 21262366 10.1016/j.neuroimage.2011.01.048

[R29] Wang Z, Bovik AC, Sheikh HR, Simoncelli EP, 2004. Image quality assessment: from error visibility to structural similarity. IEEE Trans. Image Process 13 (4), 600–612. 15376593 10.1109/tip.2003.819861

[R30] White NS, Leergaard TB, D’Arceuil H, Bjaalie JG, Dale AM, 2013. Probing tissue microstructure with restriction spectrum imaging: histological and theoretical validation. Hum. Brain Mapp 34 (2), 327–346. 23169482 10.1002/hbm.21454 PMC3538903

[R31] Xu T, Feng Y, Wu Y, Zeng Q, Zhang J, He J, Zhuge Q, 2017. A novel Richardson-Lucy model with dictionary basis and spatial regularization for isolating isotropic signals. Plos One 12 (1), e0168864. 28081561 10.1371/journal.pone.0168864 PMC5233428

[R32] Yamada N, Ueda R, Kakuda W, Momosaki R, Kondo T, Hada T, Sasaki N, Hara T, Senoo A, Abo M, 2018. Diffusion tensor imaging evaluation of neural network development in patients undergoing therapeutic repetitive transcranial magnetic stimulation following stroke. Neural Plast. 2018. 10.1155/2018/3901016 PMC5872629 29725347

[R33] Yang S, Wang J, Fan W, Zhang X, Wonka P, Ye J, 2013. An efficient ADMM algorithm for multidimensional anisotropic total variation regularization problems. Proceedings of the 19th ACM SIGKDD International Conference on Knowledge Discovery and Data Mining, pp. 641–649.

[R34] Yuh EL, Cooper SR, Mukherjee P, Yue JK, Lingsma HF, Gordon WA, Valadka AB, Okonkwo DO, Schnyer DM, Vassar MJ, , 2014. Diffusion tensor imaging for outcome prediction in mild traumatic brain injury: a track-tbi study. J. Neurotrauma 31 (17), 1457–1477. 24742275 10.1089/neu.2013.3171 PMC4144386

[R35] Zhang C, Arefin TM, Nakarmi U, Lee CH, Li H, Liang D, Zhang J, Ying L, 2020. Acceleration of three-dimensional diffusion magnetic resonance imaging using a kernel low-rank compressed sensing method. NeuroImage 210, 116584. 32004717 10.1016/j.neuroimage.2020.116584 PMC7359413

[R36] Zhang H, Schneider T, Wheeler-Kingshott CA, Alexander DC, 2012. NODDI: practical in vivo neurite orientation dispersion and density imaging of the human brain. NeuroImage 61 (4), 1000–1016. 22484410 10.1016/j.neuroimage.2012.03.072

